# Carpal Tunnel Syndrome Caused by A Fibrolipomatous Hamartoma of the Median Nerve

**DOI:** 10.1259/bjrcr.20200090

**Published:** 2021-06-22

**Authors:** Fouad Aladel, Ahmed Aldhafiri, Thabet Alghazal, Fahad Alsafran, Zainab Alrashed, Lama Karsou

**Affiliations:** 1 Departments of radiology and surgery, King Fahad Specialist Hospital in Dammam (KFSH-D), Dammam, Saudi Arabia

## Abstract

Fibrolipomatous hamartoma (FLH) of the nerve (also known as perineural lipoma, neural fibrolipoma, or lipomatosis of the nerve) is a well-known, rare benign lesion that can affect any peripheral nerve, resulting in significant enlargement of the involved nerve with fibrofatty infiltration. Although it is most commonly involving the median nerve, other peripheral nerves can be also involved. Being familiar with the pathognomonic characteristics on different imaging modalities and the association of this entity with macrodactyly help reach the diagnosis, avoid putting the patient at risk of an invasive procedure, and can guide management. We present to you a rare case of a FLH of the median nerve that was diagnosed on MRI of an adult female who presented with carpal tunnel syndrome (CTS) and progressive swelling of the right hand and wrist.

## Clinical presentation

A 36-year-old female with past medical history of kidney transplantation and hypertension was referred to our radiology department for an MRI exam, as part of the diagnostic work-up for right-hand macrodactyly and progressive swelling of the right wrist associated with carpal tunnel syndrome. Her main symptoms were pain with tingling sensation and numbness along the median nerve distribution.

**Figure 1.**
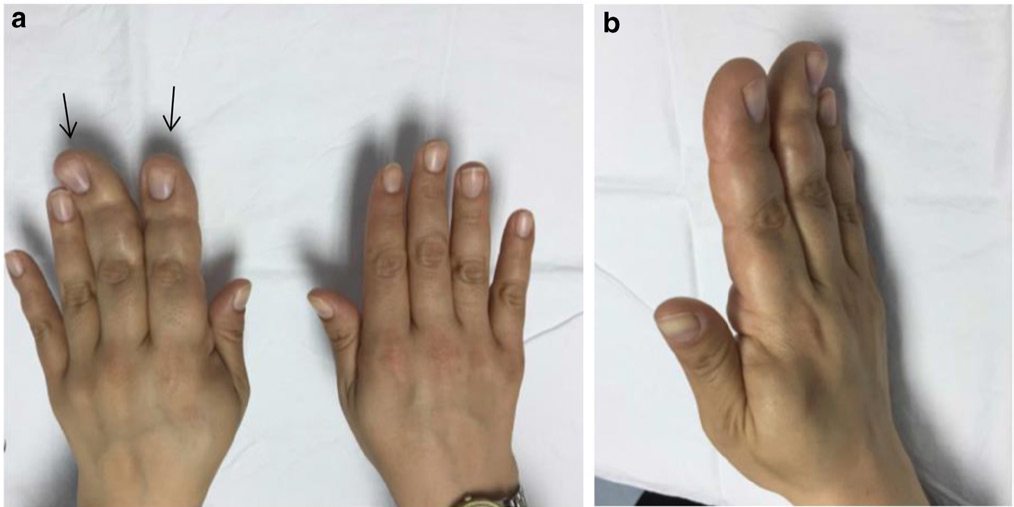
Macrodactyly of the index and the middle fingers (black arrows) of the right hand are clearly visible in comparison with the other fingers of both hands.

On physical examination, she presented with macrodactyly of the index and middle fingers of the right hand (Figure 1a) (Figure 1b) as well as volar soft tissue swelling at the level of the carpal tunnel, which was not covered on the figures. Both Tinel’s sign and Phalen’s test were positive.

## Investigations/imaging findings

**Figure 2.**
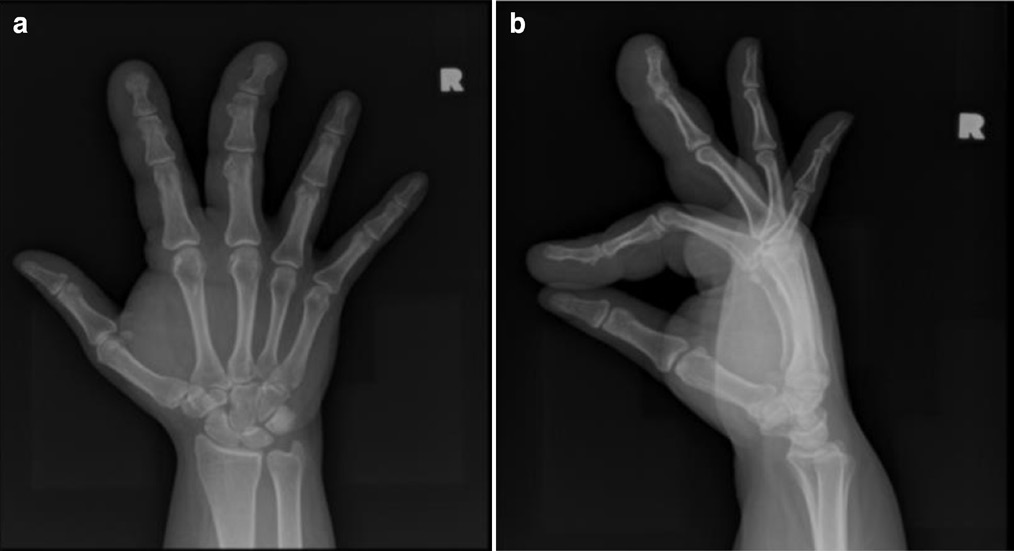
Right hand plain radiographs; a) Anteroposterior and b) Lateral views showing diffuse soft tissue swelling of the index and middle fingers with mildly enlarged phalanges compared to the other fingers.

A plain radiograph showed index and middle finger macrodactyly, manifested by asymmetric diffuse soft tissue swelling and enlargement of the phalanges compared to the other fingers (Figure 2a) and (Figure 2b).

**Figure 3.**
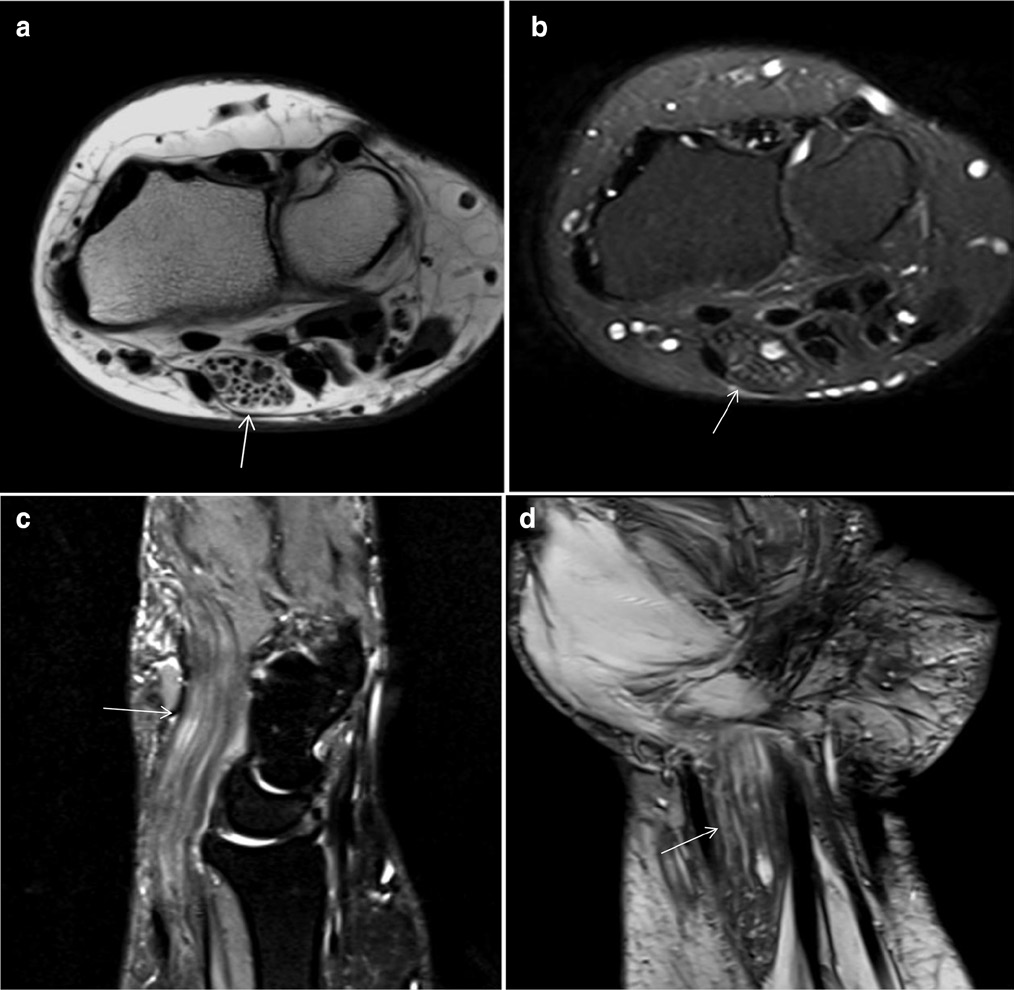
Magnetic Resonance Images a) axial T1-weighted image and b) axial T2-weighted fat saturated image showing diffuse fascicular enlargement of the median nerve with surrounding and interposing hyperintense signal on T1WI with corresponding signal loss on the T2FS-weighted images indicated fat, the so-called “co-axial cable” sign (arrow). (**c**) Magnetic resonance sagittal STIR-weighted image and d) coronal T2-weighted image showing interposition of the nerve fascicles by fatty tissue, the so-called “spaghetti” sign (arrow).

The MRI demonstrated a significantly enlarged fusiform median nerve with isointense to low signal intensity nerve fascicles on T1W and T2W images, surrounded by hyperintense fatty signal on T1W images, the “co-axial cable” sign on axial plane. It is associated with displacement of the flexor retinaculum and adjacent tendons (Figure 3a) (Figure 3b).^1^ The low T2 signal intensity nerve fascicles are separated by hyperintense fatty tissue, the “spaghetti” sign on coronal image (Figure 3d).

The final diagnosis was reached by MRI given the pathognomonic MRI appearance of fibrolipomatous hamartoma of the median nerve on MRI in combination with the provided history of macrodactyly.

## Treatment/follow-up

As FLH is a benign process, the patient was treated conservatively targeting symptomatic management of carpal tunnel syndrome without further biopsy or other invasive management.

## Discussion

FLH is characterized by separation of the axonal bundles of the nerve by mature adipocytes with subsequent thickening of the axonal bundles by perineural and endoneural fibrosis. The usually affected nerve is the median nerve representing about (66%–80%) of cases.^2^

FLH usually presents with signs and symptoms of painful nerve compression causing sensory and/or motor disturbance along the distribution of the affected nerve.

This report revealed that the patient presented with right hand macrodactyly and right wrist progressive swelling associated with tingling and numbness in the median nerve distribution. These findings were consistent with the case reported by Louaste et al as the presenting symptom was hand paresthesia in the distribution area of the median nerve with soft tissue masses noted on the distal volar aspect of the forearm with no muscle dystrophy.^3^ Also, Senger et al reported that FLH presented with swelling of the flexor aspect of wrist and thenar atrophy of the hand and positive Tinel’s test.^4^

MRI is now considered to be a diagnostic test in imaging FLH and can be definitive if the typical imaging findings are present, with no need for further investigation.^5^

The current report illustrated that MRI demonstrated significantly enlarged median nerve with low signal foci nerve fascicles surrounded by fatty signal, the “co-axial cable” sign on axial plane and separation of nerve fascicles by fatty tissue, the “spaghetti” sign on coronal plane.

Tahiri et al. at 2013 reported the same finding as MRI demonstrates a high signal intensity fusiform mass on both T1W and T2W imaging, and loss of signal on the fat suppressed images which represent the adipose proliferation. Also, the mass had the characteristic “coaxial cable-like” appearance on axial images, and a “spaghetti-like” appearance on sagittal images, which represent the circumferentially fibroid neural bundles within the fat.

Louaste et al reported that (MRI) showed a signal of fat intensity without post-gadolinium enhancement. The median nerve which was affected by the mass in its distal part of the right forearm, separating the nerve fibers, which were seen as low-intensity signal on T1W and T2W images. These features were consistent with FLH. Toms et al study illustrated that the radiological characteristics of FLH of the median nerve were considered the pathognomonic pattern of an enlarged nerve containing 15 or 16 coaxial “cables” or bundles of axons encased in fibrous tissue. The MRI findings were characteristic and pathognomonic in our case.^6^

The diagnosis of CTS is usually based on typical clinical presentation of paresthesia with or without pain in thumb through middle digits and radial aspect of ring digit. It can be confirmed with electrodiagnostic examinations in most cases.

While electrodiagnostic examinations are based on physiologic malfunctions of the median nerve, MR depicts structural abnormalities of the nerve. These MR criteria include several imaging components; median nerve swelling with abnormal cross-sectional area ratio of more than 2 mm^2^ (at the level of the pisiform and hamate respectively), bowing of the flexor retinaculum (a bowing index above 15%), increased intraneural signal on fluid-sensitive sequence, with or without nerve enhancement after T1 gadolinium administration and thenar eminence muscle atrophy in late stages.^7^

Ultrasonography is an accessible and a well-known imaging tool in diagnosis of peripheral neuropathies, in particular for CTS.^8^ However, MRI is a well-recognized imaging technique for tissue characterization and helpful for diagnosing CTS and excluding other differential diagnoses. The key imaging features of this entity and clinical associations make biopsy avoidable.

Vetrano IG et al at 2019 reported that there is a long list of FLH mimickers presenting with similar symptoms to CTS including but not limited to ganglion cyst, lipoma and Klippel-Trénaunay-Weber syndrome. Malignant peripheral nerve sheath tumours are in the list of differential diagnosis. It is important to be excluded.

A new evolving MRI technique has been reported in the literature which is diffusion tensor imaging (DTI). The principle behind it is relying on measuring water molecules diffusion along different directions by calculating fractional anisotropy (FA). In physiologic conditions, the myelin sheath allows the water molecules to move in one direction giving a high FA values. While in pathological conditions, water molecules tend to move in different directions, with subsequent reduction of FA values.

The conventional MRI techniques give a qualitative view while DTI gives quantitative information.^9^

This case shows the importance of MRI in precisely diagnosing FLH, guiding patient management and avoiding biopsy.

## Summary/Conclusion

FLH is a rare benign infiltration of the median nerve with fatty tissue associated with fibrosis. The clinical presentation is usually with swelling along the distal median nerve distribution, with or without macrodactyly and compressive median neuropathy. There is a wide differential diagnosis including benign (ganglion cyst and lipoma) and malignant entities (nerve sheath tumour with malignant potential and liposarcoma). However, imaging findings on MRI and ultrasound are pathognomonic, can exclude other entities in the differential diagnosis, and prevent further investigations. These typical MRI features were well established in our case; thus, made the final diagnosis obvious and biopsy avoidable.

## Learning points

- Diagnosis of a median nerve fibrolipomatous hamartoma (FLH) can be reached by MRI and ultrasound, without the need for further work-up or tissue sampling.
- MRI findings of a FLH of the median nerve are pathognomonic and can exclude other benign and malignant entities.
- Being familiar with the association of FLH with macrodactyly is very helpful in confidently making the diagnosis.
